# ALDH2 Suppresses Ferroptosis and Promotes Bortezomib Resistance in Multiple Myeloma Through the KEAP1‐NRF2 Feedback Loop

**DOI:** 10.1002/advs.78403

**Published:** 2026-10-28

**Authors:** Ruonan Shao, Chengcheng Liu, Xinpei Deng, Jindong Xie, Wenjian Liu, Bin Yang, Lingrui Liu, Cailu Song, Lei Li, Hua Wang, Hailin Tang

**Affiliations:** ^1^ State Key Laboratory of Oncology in South China Guangdong Provincial Clinical Research Center for Cancer Sun Yat‐sen University Cancer Center Guangzhou People's Republic of China; ^2^ Department of Hematology The Third Affiliated Hospital of Sun Yat Sen University Guangzhou People's Republic of China; ^3^ Center for Pharmacogenetics and Department of Pharmaceutical Sciences University of Pittsburgh Pittsburgh Pennsylvania USA; ^4^ Department of Pathology the First Affiliated Hospital Hengyang Medical School University of South China Hengyang People's Republic of China

**Keywords:** ALDH2, bortezomib resistance, ferroptosis, Multiple myeloma

## Abstract

Resistance to bortezomib (BTZ) represents a major obstacle that limits the therapeutic efficacy and worsens the prognosis of multiple myeloma (MM). Ferroptosis, a form of regulated cell death triggered by excessive lipid peroxidation, has garnered increasing attention for anticancer intervention, yet the mechanisms underlying ferroptosis in MM remain enigmatic. Here, we confirmed that ALDH2 is upregulated in MM patients and that its altered expression is closely associated with BTZ resistance and poor prognosis. Functionally, ALDH2 promoted the proliferation, BTZ resistance, and ferroptosis resistance of MM cells, suggesting its oncogenic role. Mechanistically, ALDH2 competitively bound to KEAP1 and stabilized NRF2 by preventing its ubiquitin‐proteasome‐mediated degradation, thereby promoting the nuclear translocation of NRF2 and subsequent upregulation of GPX4, and in turn regulating ferroptosis, proliferation, and BTZ resistance in MM. In parallel, NRF2 bound directly to the promoter region of ALDH2 and further enhanced ALDH2 transcription, thus forming a positive feedback loop. Collectively, our findings reveal that the ALDH2/KEAP1/NRF2‐GPX4 axis may serve as a key regulator of ferroptosis in MM and a promising therapeutic target for overcoming BTZ resistance.

## Graphical abstract

ALDH2, which is overexpressed in multiple myeloma, competitively binds KEAP1 to stabilize NRF2 by blocking its ubiquitin‐proteasomal degradation. Stabilized NRF2 translocates into the nucleus to upregulate the expression of GPX4. In parallel, NRF2 binds to the ALDH2 promoter to regulate its transcription, forming a positive feedback loop that represses ferroptosis and sustains bortezomib resistance.

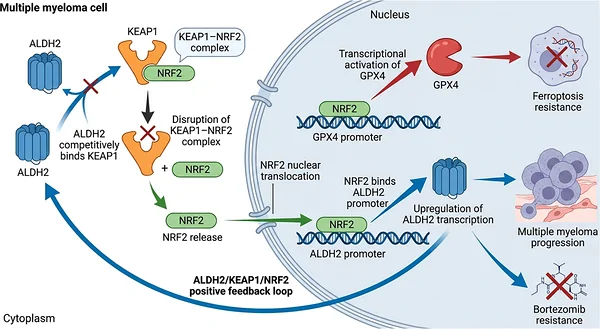

## 1 Introduction

Multiple myeloma (MM) is a hematologic malignancy marked by the clonal proliferation and infiltration of abnormal plasma cells in the bone marrow (BM) [1, 2]. As the second most common hematological malignancy, MM accounts for approximately 10% of all hematologic malignancies [3]. Patients with MM frequently suffer from osteolytic lesions, renal dysfunction, anemia, and hypercalcemia, which collectively impair the quality of life [1]. The prognosis for MM patients has significantly improved due to the widespread use of new therapeutic agents such as proteasome inhibitors, immunomodulatory drugs, and monoclonal antibodies [4]. Although proteasome inhibitors represented by bortezomib (BTZ) have markedly prolonged MM survival, unfortunately, MM remains incurable due to intrinsic or acquired resistance to BTZ [5, 6]. Therefore, elucidating the molecular mechanisms underlying BTZ resistance is critical for identifying actionable targets and improving therapeutic efficacy.

Ferroptosis is a newly recognized type of programmed cell death marked by the iron‐dependent accumulation of lipid peroxides on cell membranes [7]. Unlike apoptosis, necroptosis, or pyroptosis, ferroptosis exhibits distinct morphological features, including shrunken mitochondria with condensed membranes and reduced cristae [8, 9]. Ferroptosis is mechanistically initiated by an imbalance in redox homeostasis, where lipid peroxidation surpasses antioxidant defenses [9]. The glutathione peroxidase 4 (GPX4)‐glutathione (GSH) antioxidant system is a key defense mechanism against ferroptosis [10]. GPX4 uses GSH as its main cofactor to directly eliminate lipid peroxides, thus preventing ferroptosis [11]. Increasing evidence suggests that targeting the GPX4‐GSH axis to induce ferroptosis represents a promising strategy for cancer therapy [11, 12]. Several selective small‐molecule inhibitors targeting the GPX4‐GSH axis have been developed to trigger ferroptosis and exhibit potent antitumor effects [12]. Moreover, ferroptosis inducers have shown synergistic potential with conventional therapies, including chemotherapy, radiotherapy, and immunotherapy, in multiple tumor models [12]. Inducing ferroptosis could provide a new therapeutic strategy for addressing BTZ resistance in MM.

A variety of regulatory factors–most notably the kelch‐like ECH‐associated protein 1 (KEAP1)/nuclear factor erythroid 2‐related factor 2 (NRF2) signaling axis–orchestrate intracellular antioxidant defenses and thereby determine cellular susceptibility to ferroptosis [13]. Under physiological conditions, KEAP1 binds NRF2 in the cytoplasm, acting as a substrate adaptor for the Cullin 3‐based E3 ubiquitin ligase complex, which results in NRF2's ubiquitination and subsequent proteasomal degradation [14]. Upon oxidative stress, KEAP1 undergoes structural changes that release NRF2, enabling NRF2 to translocate into the nucleus and activate transcription of antioxidant genes–such as GPX4 and SLC7A11–via antioxidant response elements (AREs), thereby mitigating lipid peroxidation and suppressing ferroptosis [13, 15]. Recent studies have identified several regulators of the KEAP1‐NRF2 complex, such as CLK2, RNF217, TMEM160, and DPP9, which interfere with KEAP1‐mediated NRF2 degradation and enhance its transcriptional activity, ultimately modulating ferroptosis, tumor progression, and therapeutic resistance [15, 16, 17, 18]. These findings suggest that dysregulation of the KEAP1‐NRF2‐GPX4 axis may contribute to ferroptosis resistance and BTZ insensitivity in MM.

Aldehyde dehydrogenase 2 (ALDH2), a pivotal mitochondrial isoform within the aldehyde dehydrogenase superfamily, plays an essential role in detoxifying reactive aldehydes generated during oxidative stress and cellular metabolism, thereby maintaining cellular homeostasis [19]. Recent studies have implicated ALDH2 in the pathogenesis of various malignancies, where its altered expression correlates with tumor progression, poor prognosis, and chemoresistance [20, 21, 22, 23]. One previous study has confirmed that targeting ALDH2 can induce ferroptosis, thereby enhancing chemotherapy sensitivity in lung adenocarcinoma [24]. However, its functional role and clinical relevance in MM remain largely unexplored, underscoring the need for further investigation into its potential as a biomarker and therapeutic target in ferroptosis‐based interventions.

In this study, we demonstrated that ALDH2 is up‐regulated in the BM tissues of MM patients, and its altered expression is closely associated with BTZ resistance and poor prognosis. Functionally, ALDH2 promoted the proliferation, BTZ resistance, and ferroptosis resistance of MM. Mechanistically, ALDH2 competitively bound to KEAP1 and stabilized NRF2 by blocking its ubiquitin‐proteasome‐dependent degradation. This stabilization facilitated NRF2 nuclear translocation and the subsequent regulation of GPX4 expression, which in turn modulated ferroptosis, cell proliferation, and BTZ resistance in MM. In parallel, NRF2 bound directly to the promoter of ALDH2 and further enhanced ALDH2 transcription, thus forming a positive feedback loop. Overall, these findings highlight the ALDH2/KEAP1/NRF2‐GPX4 axis as a potential therapeutic target for overcoming BTZ resistance in MM.

## 2 Results

### 2.1 Elevated ALDH2 Expression Correlates with Poor Prognosis and BTZ Resistance in MM

To investigate the molecular alterations in MM, we performed mRNA‐seq using BM specimens from 3 MM patients and 3 healthy donors. Applying a filter threshold of fold‐change ≥ 1 and P‐value < 0.05, we identified 410 differentially expressed genes, among which 78 were upregulated and 332 were downregulated in MM BM tissues compared to normal controls (Figure 1A). Among these candidate genes, we noticed that ALDH2 was upregulated in MM (Figure 1A–C). ALDH2 is recognized as an oncogene in a variety of malignancies. But the possible biological function as well as the mechanism of ALDH2 in MM is elusive. Western blot analysis further confirmed the elevated expression of ALDH2 in MM BM tissues (Figure 1D). Moreover, we confirmed the overexpression of ALDH2 in MM cell lines in Figure 1E. Furthermore, qPCR analysis of 80 MM BM samples and 20 normal controls validated the upregulation of ALDH2 in MM, and its high expression was found to be associated with poor prognosis of MM (Figure 1F,G). Subsequently, we downloaded MM datasets from the GEO database and found out ALDH2 expressed highly in MM samples (Figure 1H). BTZ‐based therapies are advocated as the primary treatment for MM patients; further bioinformatic analysis revealed that elevated ALDH2 expression is correlated with BTZ resistance in MM (Figure 1I,J). Further experiments confirmed that ALDH2 protein expression was elevated in CD138+ plasma cells derived from BTZ‐resistant patients relative to BTZ‐sensitive patients (Figure S1A). To examine the association between ALDH2 expression and BTZ resistance, we continuously treated MM cell lines (U266B1 and RPMI‐8226) by incrementally increasing the concentration of BTZ every 1–2 weeks until BTZ‐resistant MM cell lines (U266B1R and RPMI‐8226R) were established. Subsequently, we measured ALDH2 expression in BTZ‐susceptible and BTZ‐resistant MM cell lines. qPCR and western blot demonstrated a significant upregulation of ALDH2 mRNA and protein levels in BTZ‐resistant MM cells compared to BTZ‐susceptible MM cells (Figure S1B,C). These findings suggest that ALDH2 is overexpressed in both MM patients and BTZ‐resistant cell lines.

**FIGURE 1:**
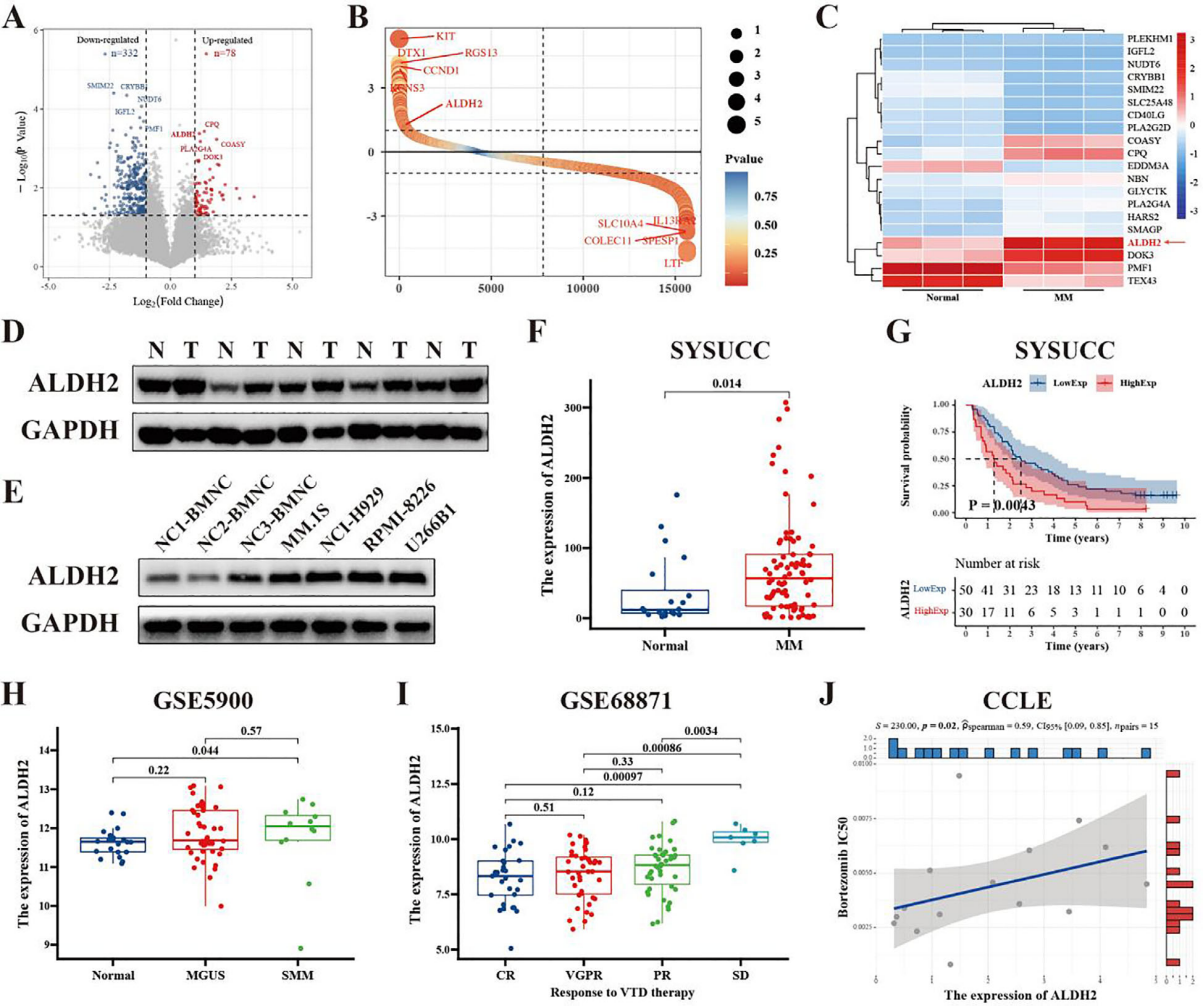
ALDH2 correlates with poor prognosis and BTZ resistance in MM. (A) Volcano plot showing differentially expressed genes based on absolute fold change >1 and FDR < 0.05. (B) Gene rank based on fold change. (C) Heatmap indicating the top 10 upregulated and downregulated genes in BM specimens from 3 MM patients and 3 healthy donors. (D) ALDH2 levels in BM specimens, including 5 normal samples and 5 MM samples detected by western blot. (E) ALDH2 protein levels were higher in MM cells than those of bone marrow mononuclear cells (BMNC) from healthy donors. (F) ALDH2 levels in BM specimens, including 20 normal samples and 80 MM samples, detected by qPCR. (G) Kaplan‐Meier survival analysis of OS for 80 MM patients with high vs. low ALDH2 expression in the SYSUCC cohort. (H) Expression signatures of ALDH2 in the GSE5900 cohort. (I) High expression of ALDH2 was correlated with poor chemotherapy response in the GSE68871 cohort. (J) Elevated ALDH2 expression is associated with increased IC50 values of BTZ in the CCLE dataset.

### 2.2 ALDH2 Facilitates the Proliferation of MM

To investigate the potential role of ALDH2 in MM, two siRNAs targeting ALDH2 and an overexpression plasmid were employed to modulate its expression in MM cells. Subsequent qPCR and western blot analyses confirmed significant downregulation or overexpression of ALDH2 in MM cells following transfection with siRNAs or overexpression plasmids, respectively (Figure 2A,B). We conducted CCK‐8, EdU, and soft agar colony formation assays to assess MM cell viability. The study found that overexpressing ALDH2 significantly enhanced the proliferation of MM cells, whereas knocking down ALDH2 markedly inhibited their proliferation (Figure 2C–F). The findings indicate that ALDH2 functions as an oncogene in MM.

**FIGURE 2:**
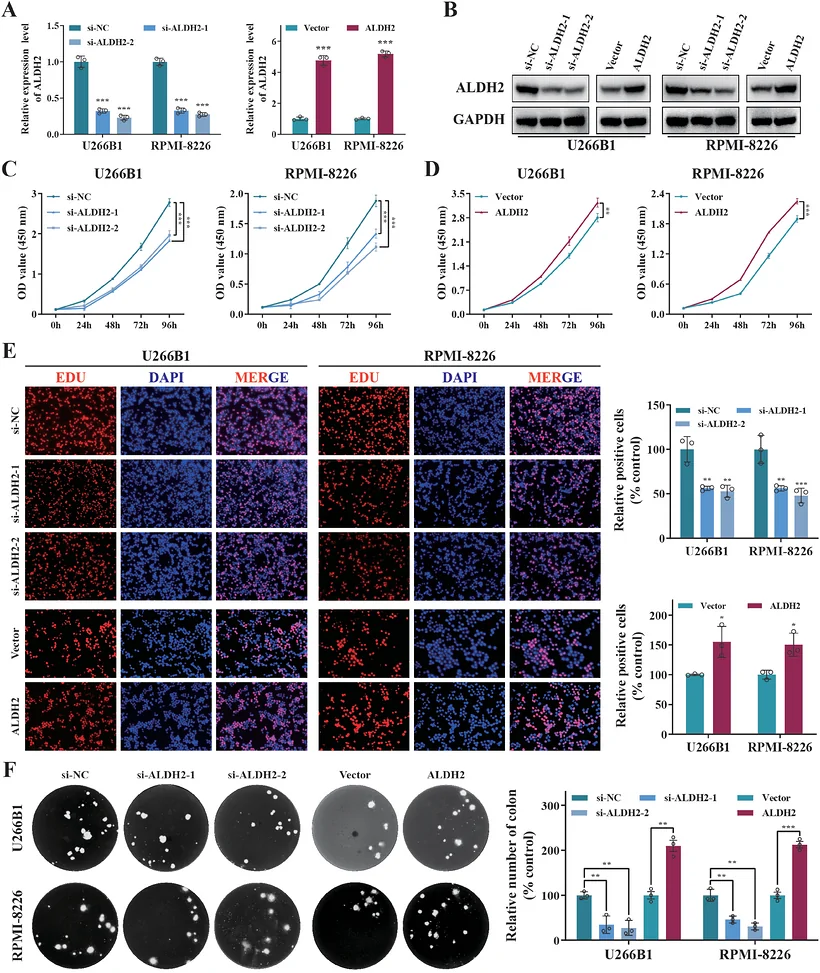
ALDH2 promotes the proliferation of MM. (A, B) qPCR and Western blot were performed to measure the efficiency of overexpression and knockdown of ALDH2. (C, D) CCK‐8 assay for analyzing the growth curves of MM cells. (E) EdU assays evaluating the proliferation of MM cells (Scale bar = 50 µm). (F) Soft agar colony formation assay evaluating the cell proliferation ability. The data are shown as the mean ± SD; ^*^ *p* < 0.05, ^**^ *p* < 0.01, ^***^ *p* < 0.001, and all above experiments have been repeated three times.

### 2.3 ALDH2 Serves as a Key Mediator of BTZ Resistance in MM

We explored ALDH2's role in BTZ resistance by evaluating the IC50 of BTZ in MM cells after altering ALDH2 expression. The cell viability curve indicated that ALDH2 downregulation notably reduced the IC50 value of BTZ, while its upregulation elevated the IC50 value in MM cells. These findings implicate ALDH2 as a potential mediator of BTZ resistance in MM (Figure 3A,B). Flow cytometry analysis demonstrated a significantly increased apoptosis rate in ALDH2 knockdown MM cells compared to the control group under BTZ treatment (Figure 3C). This finding suggests that the down‐regulation of ALDH2 has the potential to enhance the susceptibility of MM cells to BTZ. We generated BTZ‐resistant MM cell populations, U266B1R and RPMI‐8226R. Knockdown of ALDH2 significantly reversed the BTZ‐resistance phenotype in BTZ‐resistant MM cells (Figure 3D,E). We utilized mice xenograft models treated with BTZ to validate ALDH2's role in chemosensitivity in vivo. After 4 weeks of treatment, tumors in the ALDH2 overexpression group were significantly larger and heavier than those in the control group. Correspondingly, ALDH2 overexpression attenuated the effect of BTZ (Figure 3F–I). These findings indicate that ALDH2 significantly contributes to BTZ resistance in MM cells. This conclusion aligns with the results mentioned in Figure 1I,J and Figure S1.

**FIGURE 3:**
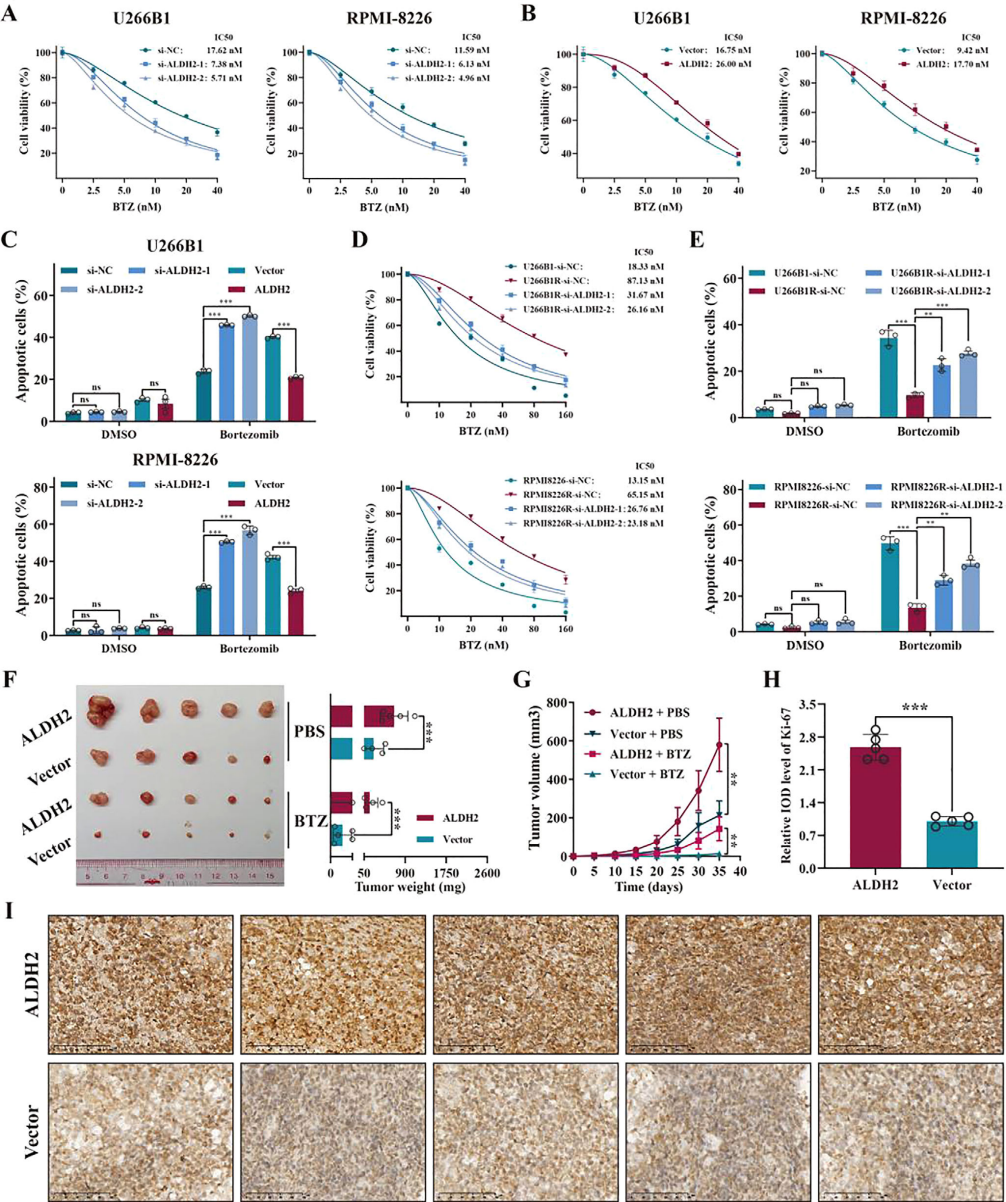
ALDH2 promotes BTZ resistance in MM cells. (A, B) Dose‐response curves of BTZ in MM cells measured by CCK‐8 assay. (C) The indicated cells were treated with BTZ (10 nm) or DMSO (control) and subjected to Annexin FITC/PI stanning to detect apoptotic rates by flow cytometry. (D) Dose‐response curves of BTZ in U266B1R and RPMI‐8226R cells measured by CCK‐8 assay. (E) The U266B1R and RPMI‐8226R cells were treated with BTZ (10 nm) or DMSO (control) and subjected to Annexin FITC/PI stanning to detect apoptotic rates by flow cytometry. (F) ALDH2 overexpression impaired the BTZ effects of MM in vivo. Left panel, representative image of tumors from xenografted mice treated with PBS or BTZ after 4 weeks (*n* = 5 each group); right panel, the weight of tumors was measured after surgical dissection. (G) The volumes of tumors were measured every 5 days, and the growth curves of each group were drawn. (H) Representative H&E staining of the tumor tissues was shown. (I) IHC staining images (Ki‐67) of tumor tissues. The data are shown as the mean ± SD, ^**^ *p* < 0.01, ^***^ *p* < 0.001, and all above experiments have been repeated three times.

### 2.4 ALDH2 Functions as a Key Regulator of Ferroptosis in MM

We conducted RNA sequencing on ALDH2‐knockdown and control U266B1 cells to elucidate ALDH2's role in MM proliferation and BTZ resistance. A total of 328 genes exhibited downregulated expression, while 186 genes showed upregulated expression, determined by a fold change ≥ 1 and an FDR < 0.05, as illustrated in the volcano plot (Figure S2A,B). GSEA enrichment analysis was conducted on differentially expressed genes. Interestingly, we found that ALDH2 was greatly associated with Reactive oxygen species (ROS) metabolism, a pathway fundamentally involved in ferroptosis (Figure 4A). Emerging evidence suggests that ferroptosis is integral to the pathophysiology of MM [25, 26]. MM cells are particularly vulnerable to the ferroptosis inducer RSL3, highlighting the potential of exploiting this metabolic weakness as a new therapeutic strategy for MM [25]. However, the role of ALDH2 in ferroptosis within MM remains unexplored. Therefore, we undertook experimental investigations to clarify the potential link between ALDH2 and ferroptosis. The cell viability curve indicated that ALDH2 knockdown increased the sensitivity of MM cells to RSL3. Correspondingly, ALDH2 overexpression attenuated the effect of RSL3 (Figure 4B). RSL3‐induced cell death and MDA accumulation significantly increased following ALDH2‐knockdown, but were effectively mitigated by the ferroptosis inhibitor ferrostatin‐1 (Fer‐1), whereas the apoptosis inhibitor Z‐VAD‐FMK (Z‐V‐F), necroptosis inhibitor Necrostatin‐1 (Nec‐1), and autophagy inhibitor Bafilomycin‐A1 (Baf1) showed no such effect (Figure 4C,D and Figure S2C). Furthermore, the application of C11‐BODIPY probe staining unveiled a noteworthy increase in the cytomembrane lipid peroxidation accumulation rate induced by RSL3 after ALDH2‐knockdown and reversed by fer‐1 (Figure 4E). In contrast, ALDH2 overexpression yields the opposite outcome (Figure 4C–E). We utilized mice xenograft models treated with RSL3 to verify ALDH2's role in ferroptosis in vivo. After a 4‐week treatment, the ALDH2 overexpression group exhibited a significant increase in tumor size and weight compared to the control group. Correspondingly, ALDH2 overexpression attenuated the effect of RSL3 (Figure 4F–H). MDA and 4‐HNE accumulation was remarkably reduced after ALDH2 overexpression (Figure 4I,J). These findings indicate that ALDH2 plays a crucial role in regulating ferroptosis in MM.

**FIGURE 4:**
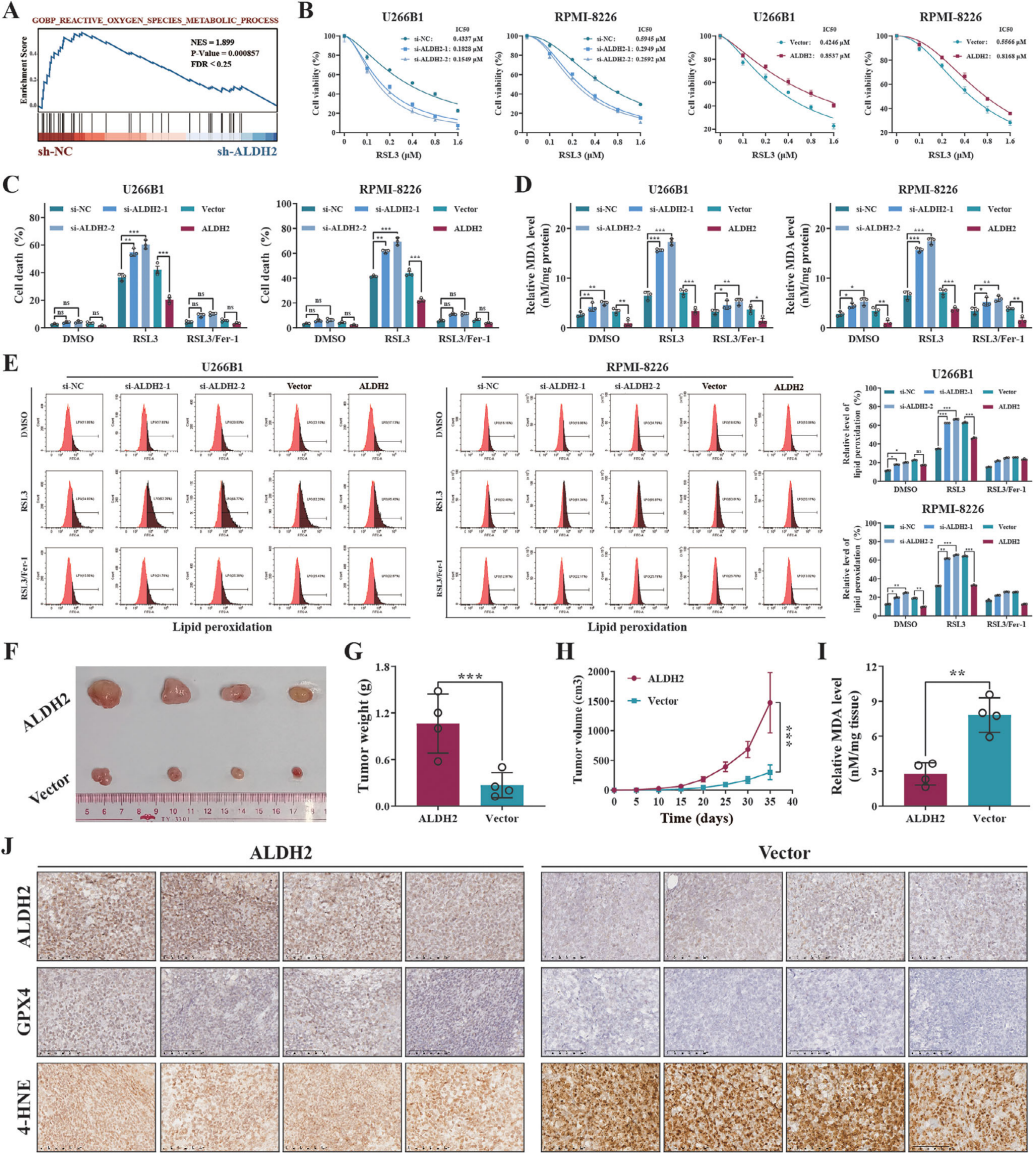
Knockdown of ALDH2 Sensitizes MM Cells to Ferroptosis. (A) GSEA displaying correlations between ALDH2 expression and biological process in MM cells. (B) Dose‐response curves of RSL3 (0, 0.1, 0.2, 0.4, 0.8, 1.6 µm) in MM cells measured by CCK‐8 assay. (C) Cell death was assessed through flow cytometry in MM cells exposed to RSL3 (0.5 µm) in combination with Fer‐1 (10 µm). (D) The cellular MDA levels were increased after RSL3 treatment (0.5 µm) and rescued by the presence of Fer‐1 (10 µm). (E) Lipid peroxidation was measured by BODIPY 581/591 C11 staining in MM cells subjected to RSL3 (0.5 µm) in combination with Fer‐1 (10 µm). (F) Representative image of tumors from xenografted mice treated with RSL3 after 4 weeks (*n* = 4 each group). (G) The weight of tumors was measured after surgical dissection. (H) The volumes of tumors were measured every 5 days, and the growth curves of each group were drawn. (I) The MDA levels in tumor tissues were quantified after surgical dissection. (J) IHC staining images (ALDH2, GPX4, and 4‐HNE) of tumor tissues. The data are shown as the mean ± SD, ^ns^p>0.05, ^*^ *p* < 0.05, ^**^ *p* < 0.01, ^***^ *p* < 0.001, and all above experiments have been repeated three times.

### 2.5 ALDH2 Knockdown Attenuates BTZ Resistance of MM Cells by Inducing Ferroptosis

To explore the mechanisms behind BTZ resistance in MM, we examined if ALDH2 depletion enhances BTZ treatment efficacy on BTZ‐resistant cell death by inducing ferroptosis. In BTZ‐exposed U266B1R and RPMI‐8226R cells, pharmacological inhibition of ferroptosis by Fer‐1 effectively rescued cell death caused by ALDH2 knockdown. While the apoptosis inhibitor Z‐V‐F produced only a modest protective effect against cell death, blockade of autophagy with Baf1 showed no rescuing activity (Figure S3A).

To validate this observation, we evaluated intracellular lipid peroxidation and Cleaved‐caspase‐3 levels in ALDH2‐depleted U266B1R and RPMI‐8226R cells treated with BTZ. ALDH2 knockdown resulted in a marked elevation of intracellular lipid peroxidation levels, whereas only a modest increase in Cleaved‐caspase‐3 activity was observed (Figure S3B,C). As demonstrated by flow cytometry, the combination of BTZ and RSL3 significantly increased intracellular lipid peroxidation levels and MDA accumulation in MM cells (Figure S3D,E). Furthermore, Co‐treatment of BTZ and RSL3 synergized in inhibition of cell viability. The combination of the two drugs synergistically inhibited cell viability at CI values < 1 (Figure S3F). These findings indicate that ALDH2 knockdown enhances BTZ sensitivity in MM cells by triggering ferroptosis.

### 2.6 ALDH2 Exerts Biological Function by Regulating the Expression of GPX4

To further elucidate the molecular mechanism underlying ALDH2‐mediated ferroptosis, we analyzed the RNA‐Seq data in Figure S2A,B and found that the key ferroptosis regulator GPX4 was markedly downregulated following ALDH2 knockdown (Figure 5A). Bioinformatic analysis and western blot revealed that GPX4 expression is positively correlated with ALDH2 expression (Figure 5B,C). Moreover, we confirmed the upregulation of GPX4 in MM, and its high expression was found to be associated with poor prognosis of MM (Figure 5D,E). Overexpression of ALDH2 led to a significant increase in GPX4 expression, whereas knockdown of ALDH2 resulted in a notable decrease in GPX4 expression (Figure 5F). GPX4 overexpression rescued the impaired proliferation, BTZ tolerance, and ferroptosis resistance caused by ALDH2 knockdown in MM cells (Figure 5G–K and Figure S4). These observations collectively indicate that ALDH2 influences biological function through the regulation of GPX4 expression.

**FIGURE 5:**
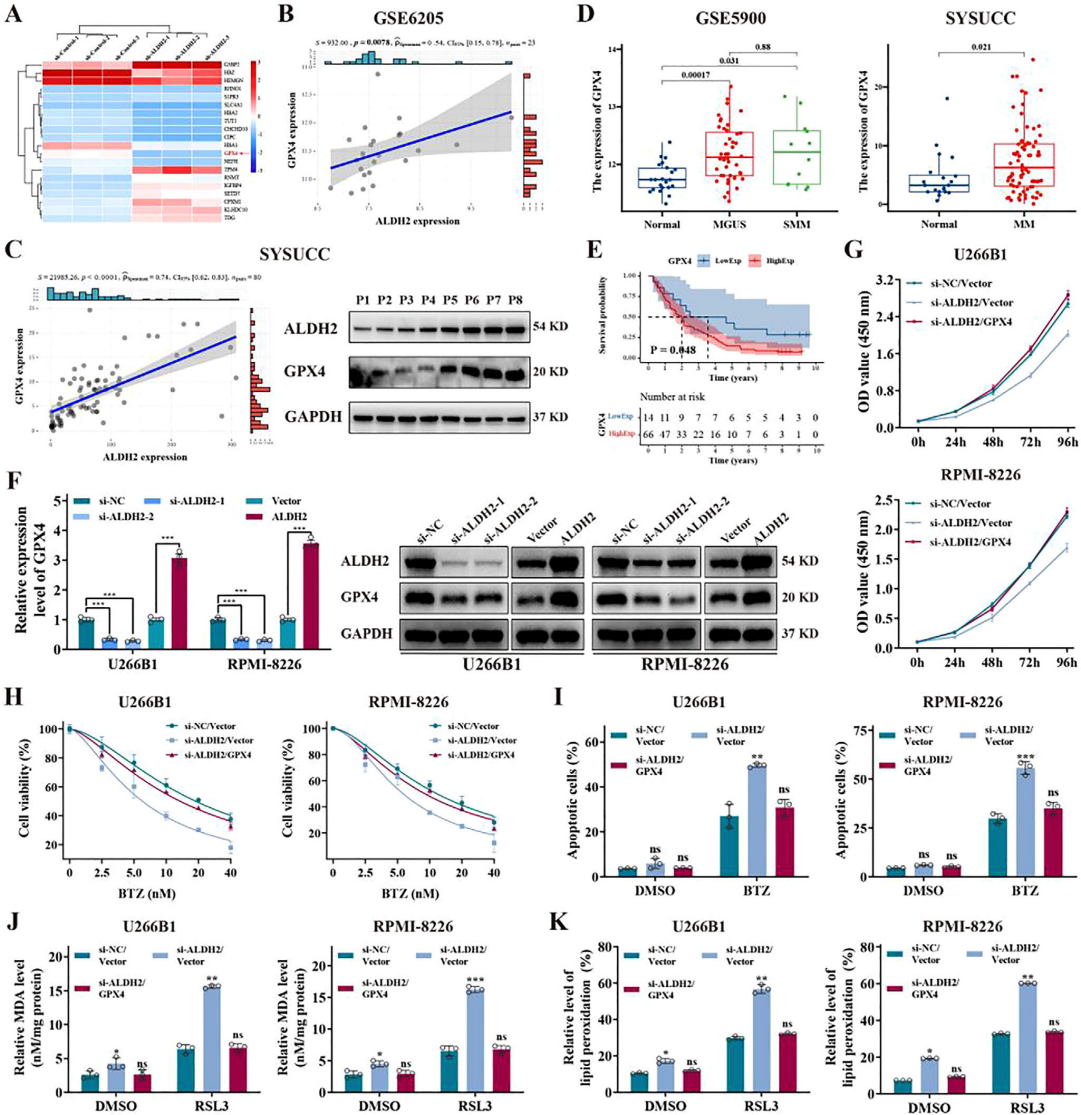
ALDH2 regulates GPX4 expression in MM. (A) Heatmap indicating the top 10 upregulated and downregulated genes between si‐NC (negative control) and si‐ALDH2 cells as detected by RNA next‐generation sequencing with three biological duplicates. (B) Correlation between GPX4 and ALDH2 expression in the GSE6205 cohort. (C) The correlation between GPX4 and ALDH2 expression was examined in the SYSUCC cohort. (D) Expression signatures of GPX4 in the GSE5900 cohort and SYSUCC cohort. (E) GPX4 levels in BM specimens, including 20 normal samples and 80 MM samples detected by qPCR. (E) Kaplan‐Meier survival analysis of OS for 80 MM patients with high vs. low GPX4 expression in the SYSUCC cohort. (F) After knockdown or overexpression of ALDH2, the change in GPX4 expression level was analyzed. (G) CCK‐8 assay for analyzing the growth curves of MM cells. (H) Dose‐response curves of BTZ in MM cells measured by CCK‐8 assay. (I) The indicated cells were treated with BTZ (10 nm) or DMSO (control) and subjected to Annexin FITC/PI stanning to detect apoptotic rates by flow cytometry. (J, K) MDA and Lipid peroxidation were measured in MM cells subjected to RSL3 (0.5 µm) treatment. The data are shown as the mean ± SD, ^ns^p>0.05, ^*^ *p* < 0.05, ^**^ *p* < 0.01, ^***^ *p* < 0.001, and all above experiments have been repeated three times.

### 2.7 ALDH2 Upregulates GPX4 Expression via KEAP1/NRF2 Axis

To elucidate the underlying mechanism through which ALDH2 regulates GPX4 expression, Co‐IP assay followed by mass spectrometry and silver staining were performed to identify proteins that potentially interact with ALDH2 in MM cells. The specific band was identified to be KEAP1 through mass spectrometry (Figure 6A and Figure S5A,B). As we know, KEAP1 serves as a substrate adaptor for the E3 ubiquitin ligase complex, targeting NRF2 for rapid ubiquitin‐proteasome degradation and preventing its nuclear translocation and transcriptional activation [14]. NRF2, a pivotal regulator of cellular antioxidant defense, drives the transcription of genes such as GPX4, SLC7A11, and HO‐1, thereby suppressing ferroptosis [13, 15]. We hypothesize that ALDH2 upregulates GPX4 expression via the KEAP1/NRF2 axis. To further delineate the interplay between ALDH2 and the KEAP1/NRF2 axis, we assessed KEAP1 and NRF2 expression after ALDH2 knockdown or overexpression. Modulation of ALDH2 expression did not affect KEAP1 mRNA or protein levels, nor did it alter NRF2 mRNA levels. However, NRF2 protein levels were decreased in ALDH2‐deficient cells and increased in ALDH2‐overexpressing cells (Figure 6B,C). Our results indicate that ALDH2 may exert its function by stabilizing NRF2 protein and promoting its nuclear translocation, which in turn regulates GPX4 expression. As expected, NRF2 overexpression rescued the impaired GPX4 protein levels caused by ALDH2 knockdown, and NRF2 knockdown also led to a decrease in GPX4 protein levels in MM cells (Figure 6D). Subsequently, we conducted experiments to validate the impact of ALDH2 on NRF2 nuclear translocation. We found that ALDH2 knockdown markedly attenuated NRF2 nuclear translocation, whereas ALDH2 overexpression restored this process. Furthermore, treatment with the NRF2 activator tBHQ significantly enhanced NRF2 nuclear translocation. Notably, tBHQ‐induced NRF2 nuclear translocation was also associated with an increase in ALDH2 protein levels, suggesting a reciprocal regulatory relationship between ALDH2 and NRF2 (Figure 6E). Consistently, immunofluorescence confirmed that ALDH2 knockdown diminished NRF2 nuclear localization (Figure 6F). These findings indicate that ALDH2 upregulates GPX4 expression via the KEAP1/NRF2 axis.

**FIGURE 6:**
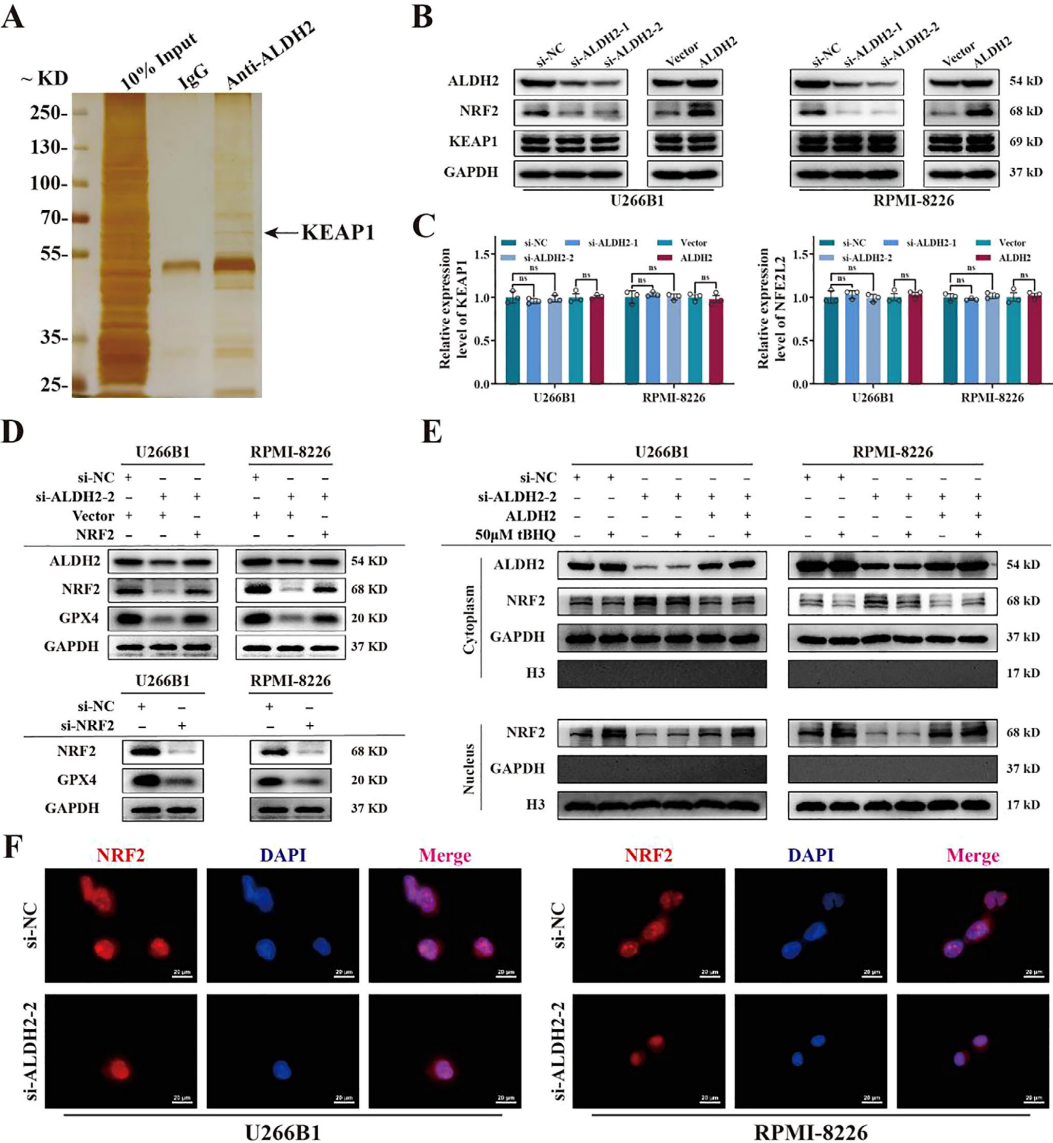
ALDH2 enhanced GPX4 expression through the KEAP1/NRF2 signaling pathway. (A) The immunoprecipitation assay was carried out using an ALDH2 antibody. Samples were separated by silver staining followed by mass spectroscopy in U266B1 cells. (B, C) After knockdown or overexpression of ALDH2, the changes in KEAP1 and NRF2 expression levels were analyzed. (D) After knockdown or overexpression of NRF2, the change in GPX4 expression level was analyzed. (E) Knockdown of ALDH2 increased the nuclear translocation of NRF2, and this effect could be rescued by overexpression of ALDH2 or tBHQ treatment. (F) Immunofluorescence was used to localize NRF2 in cells with or without ALDH2 knockdown. MM cells were labeled with anti‐NRF2 (Red) and DAPI (blue). Scale bar: 20 µm. The data are shown as the mean ± SD, ^ns^p>0.05, and all above experiments have been repeated three times.

### 2.8 ALDH2 Prevents the Degradation of NRF2 Protein

We hypothesized that ALDH2 stabilizes NRF2 protein by preventing its degradation. Accordingly, we treated MM cells with the protein synthesis inhibitor, cycloheximide (CHX), and analyzed the degradation rate of NRF2. Indeed, we found that the degradation of NRF2 was accelerated upon ALDH2 knockdown in MM cells (Figure 7A,B). To investigate whether ALDH2 knockdown accelerated NRF2 degradation by increasing its ubiquitination via the ubiquitin‐proteasome pathway, MM cells were treated with the proteasome inhibitor MG132. MG132 treatment successfully restored NRF2 protein levels in ALDH2‐deficient MM cells (Figure 7C). A ubiquitination assay revealed that ALDH2‐knockdown elevated basal levels of ubiquitin‐conjugated NRF2, while ALDH2 overexpression decreased these levels in MM cells (Figure 7D). These findings indicate that ALDH2 may stabilize NRF2 by indirectly preventing its degradation via the ubiquitin‐proteasome pathway.

**FIGURE 7:**
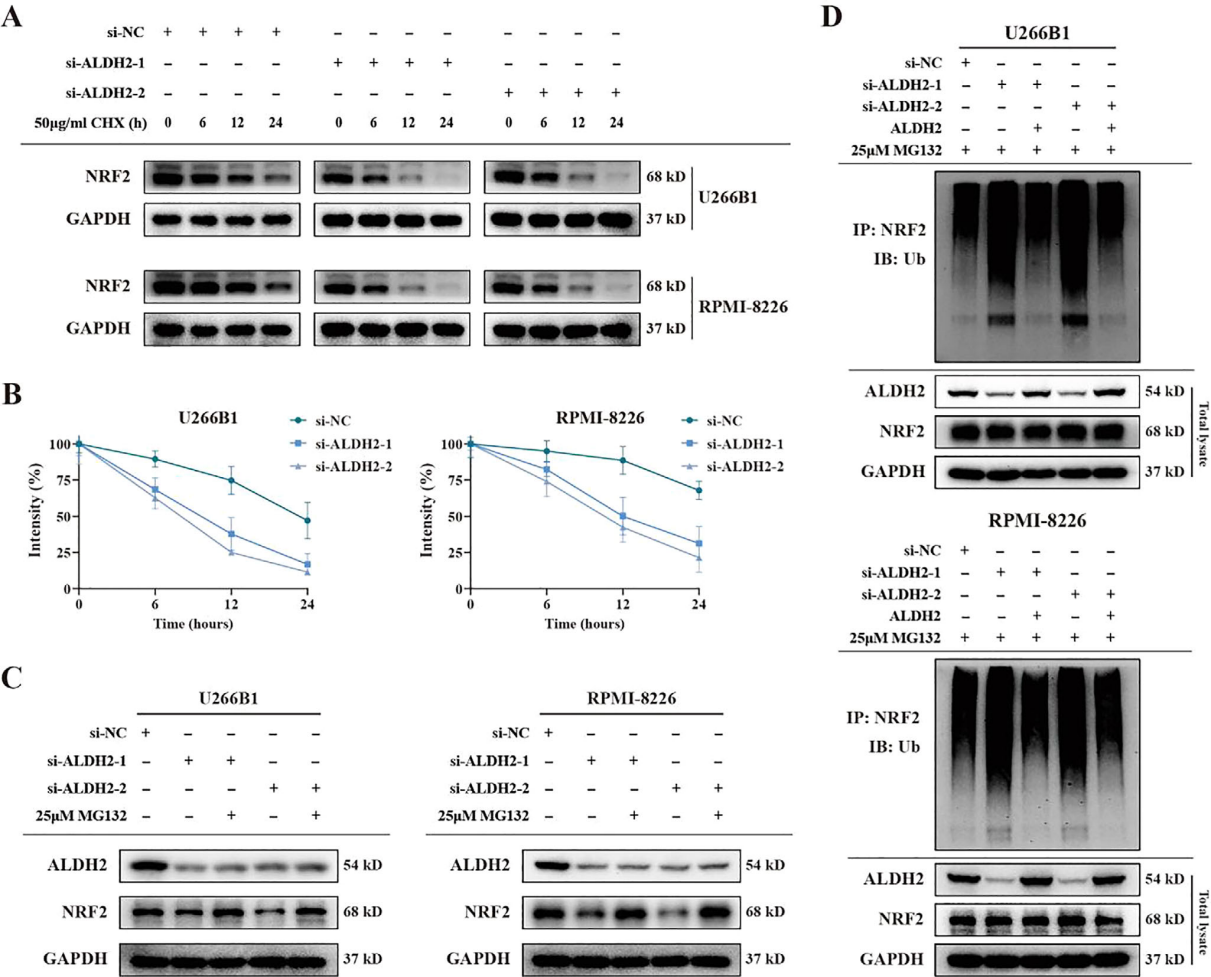
ALDH2 protects NRF2 from ubiquitin‐proteasome degradation. (A) ALDH2 stabilized NRF2 under basal conditions. MM cells transfected with ALDH2 knockdown and control plasmids were left untreated or treated with CHX (50 µg/mL) and incubated for the indicated time periods. Lysates were analyzed by Western blotting. (B) Statistical quantitation of the NRF2 protein intensity. (C) ALDH2 reduced the protein degradation of NRF2. MM cells transfected with ALDH2‐knockdown and control plasmids were left untreated or treated with 25 µm of MG132 for 4 h to block the degradation of ubiquitinated proteins. (D) ALDH2 reduced the ubiquitination of NRF2. Control or ALDH2‐knockdown MM cells were transfected with or without ALDH2 overexpression plasmids, treated with 25 µm of MG132 for 4 h, and subjected to an in vivo ubiquitination assay to detect Ubiquitin‐conjugated endogenous NRF2 proteins. Lysates were denatured, immunoprecipitated with anti‐NRF2, and blotted with an anti‐Ubiquitin antibody.

### 2.9 ALDH2 Competes with NRF2 for KEAP1 Binding

To further investigate the mechanism by which ALDH2 prevents the degradation of NRF2 protein, Co‐IP analysis was performed and revealed that ALDH2 does not directly interact with NRF2 (Figure 8A,B). KEAP1 is well known to act as a substrate adaptor to bring NRF2 into the Cul3‐dependent E3 ubiquitin ligase complex, resulting in the rapid proteasome‐mediated degradation of NRF2 [15]. Our study revealed that ALDH2 interacts with KEAP1, yet alteration in ALDH2 expression did not influence KEAP1 mRNA and protein levels. Importantly, ALDH2 prevents the degradation of NRF2 protein. Taken together, we hypothesize that ALDH2 may compete with NRF2 for KEAP1 binding, thereby preventing NRF2 from ubiquitin‐proteasome‐mediated degradation (Figures 6 and 7). Accumulating evidence indicates that two separate sequences, the DLG and E(S/T)GE motifs, are responsible for binding to KEAP1. Recent reports have shown that some proteins can compete with NRF2 for binding to KEAP1 by inhibiting the proper binding of the DLG or E(S/T)GE motifs [15]. For example, the p62/SQSTM1 protein binds to KEAP1 by the linear sequence, STGE, which resembles the ETGE sequence that NRF2 uses to interact with the Kelch domain of KEAP1 [15]. Using the Eukaryotic Linear Motif (ELM, http://elm.eu.org/search.html) database, we detected a putative STGE short‐linear motif in the human ALDH2 sequence, which matches the ELM degron class DEG_Kelch_keap1_1 for KEAP1 recognition (Figure S5C). Further structural inspection of the experimentally‐determined human ALDH2 crystal structure (PDB: 1O1H) retrieved from the RCSB Protein Data Bank (RCSB‐PDB) database showed that this STGE‐containing segment is solvent‐exposed on the protein surface rather than buried within the interior of ALDH2 (Figure S5D). These observations raise the possibility that ALDH2 may bind KEAP1 via this segment and compete with NRF2 for KEAP1 engagement (Figure S5E). Furthermore, Co‐IP analysis substantiated that ALDH2 directly bound to KEAP1, thereby reinforcing the evidence for their molecular association (Figure 8C,D). Immunofluorescence analysis demonstrated the colocalization of KEAP1 and ALDH2 across the cells (Figure 8E). In MG132‐treated MM cells, Co‐IP assays revealed increased binding of KEAP1 to ubiquitinated NRF2 following ALDH2 knockdown (Figure 8F and Figure S5F). Further experiments revealed that overexpression of KEAP1 could restore the weakened binding of KEAP1 to ubiquitinated NRF2 caused by overexpression of ALDH2 (Figure S5G). The findings indicate that ALDH2 competitively binds to KEAP1, inhibiting the KEAP1‐NRF2 interaction and subsequent degradation of NRF2.

**FIGURE 8:**
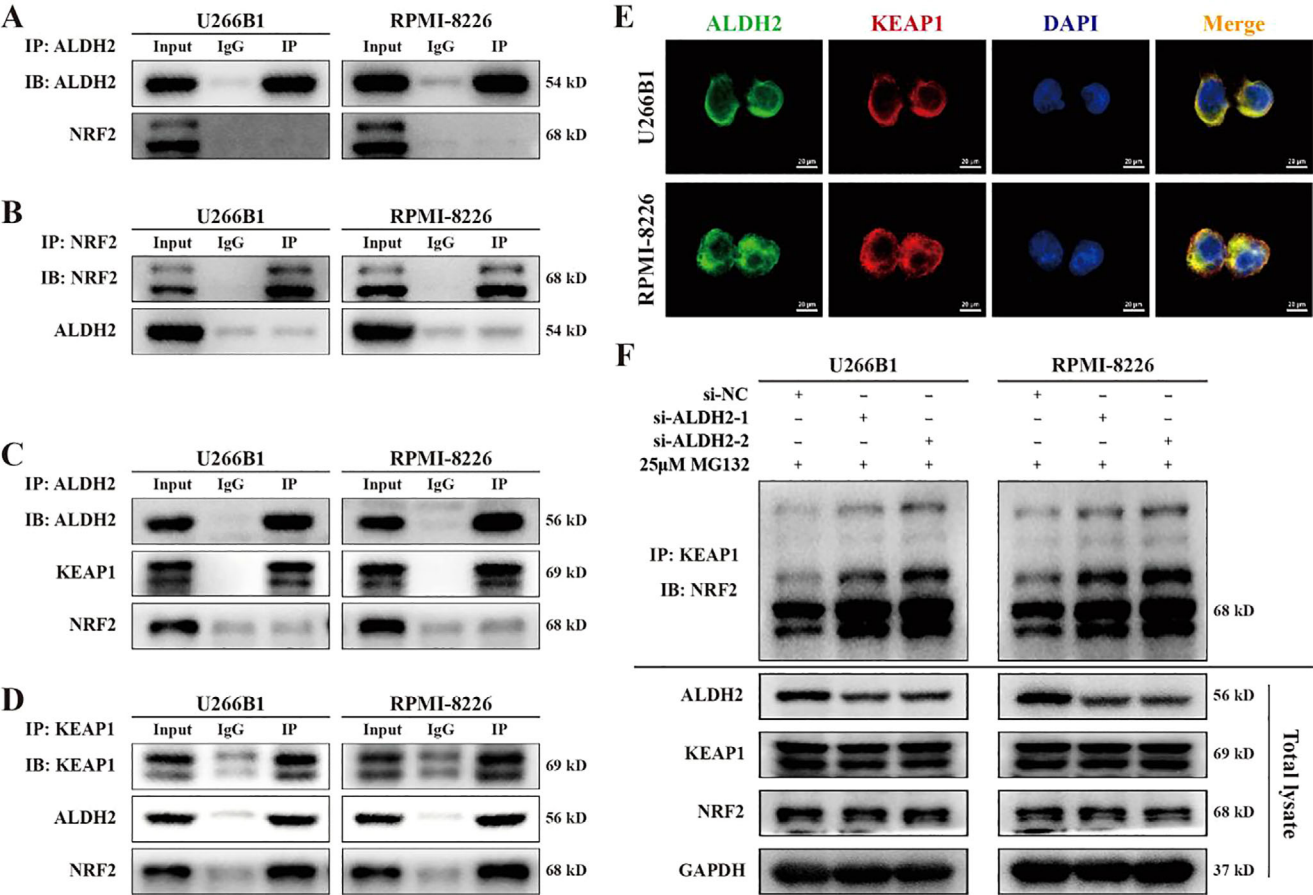
ALDH2 interferes with the KEAP1‐dependent ubiquitination of NRF2 by competitively binding to KEAP1. (A) The immunoprecipitation was performed using an ALDH2 antibody, and then blotted for NRF2. (B) The immunoprecipitation was performed using an NRF2 antibody, and then blotted for ALDH2. (C) The immunoprecipitation was performed using an ALDH2 antibody, and then blotted for KEAP1 and NRF2. (D) The immunoprecipitation was performed using a KEAP1 antibody, and then blotted for ALDH2 and NRF2. (E) The localizations of endogenous KEAP1 and ALDH2 in MM cells were determined by double‐label indirect immunofluorescence with anti‐KEAP1 (red) and anti‐ALDH2 (green) antibodies. The colocalization of KEAP1 and Nestin is indicated by a yellow color in the merged images. Scale bar: 20 µm. (F) ALDH2 reduced the interaction between NRF2 and KEAP1. Control or ALDH2‐knockdown MM cells were treated with 25 µm MG132 for 4 h. Cell lysates were immunoprecipitated with an anti‐KEAP1 antibody and blotted with an anti‐NRF2 antibody.

### 2.10 NRF2 Promotes the Transcription of ALDH2

Notably, the above data revealed that the NRF2 activator tBHQ increased the expression of ALDH2 in MM cells, supporting the notion that NRF2 may participate in its transcriptional regulation (Figure 6E). JASPAR database (https://jaspar.elixir.no/) supports this hypothesis by identifying a potential ARE sequence in the ALDH2 promoter region that NRF2 could recognize (Figure 9A). ChIP analysis in MM cells, followed by qPCR, confirmed that NRF2 directly binds to the ARE sequences in the ALDH2 promoter, indicating its regulatory role in ALDH2 transcription (Figure 9B). The promoter dual‐luciferase reporter assay demonstrated that transfection with the NRF2 overexpression plasmid significantly enhanced luciferase activity in the ARE‐Wt construct, while the ARE‐Mut construct exhibited no notable change, confirming the functional importance of the predicted ARE site (Figure 9C,D). Aligned with these findings, tBHQ treatment elevated the mRNA and protein expression levels of ALDH2 and NRF2‐downstream genes (Figure 9E,F). NRF2‐knockdown downregulated the expression of ALDH2 and NRF2‐downstream genes (Figure 9G,H). Furthermore, the protein levels of NRF2, ALDH2, and GPX4 in BTZ‐resistant MM cells were significantly upregulated compared to BTZ‐susceptible MM cells (Figure S6A). To examine whether ALDH2‐driven NRF2 activation is self‐reinforcing, we generated doxycycline (Dox)‐inducible ALDH2‐overexpression cells and followed ALDH2 and NRF2 protein levels across a time course of induction in the presence or absence of the NRF2 inhibitor ML385. Dox treatment produced a progressive accumulation of ALDH2 that was accompanied by a parallel, time‐dependent rise in NRF2 protein (Figure S6B). Co‐treatment with ML385 did not abolish this response but consistently blunted it: NRF2 still increased over time, yet the magnitude of accumulation was modestly and reproducibly lower at each time point than in vehicle‐treated cells (Figure S6B). Together, these data indicate that ALDH2 elevates NRF2 primarily through protein stabilization, and that the resulting NRF2 transcriptional activity further amplifies its own accumulation, consistent with a self‐amplifying NRF2 module operating downstream of ALDH2. Next, we explored NRF2's role in BTZ resistance by evaluating the IC50 of BTZ in MM cells after altering NRF2 expression. The cell viability curve indicated that NRF2 downregulation notably reduced the IC50 value of BTZ, while its upregulation elevated the IC50 value in MM cells (Figure S6C). Furthermore, Co‐treatment of BTZ and ML385 synergized in inhibiting cell viability. The combination of the two drugs synergistically inhibited cell viability at CI values < 1 (Figure S6D). To further confirm the role of ML385 in BTZ‐mediated anti‐MM activity in vivo, we constructed a U226B1 cell xenograft mouse model to evaluate the anti‐tumor efficacy of ML385 and BTZ through mono‐ or combination therapies. Our results indicated that ML385 treatment potently enhanced the MM growth inhibition induced by BTZ monotherapy (Figure S6E,F). These findings indicate that ALDH2 is a target gene of NRF2, which enhances ALDH2 transcription and expression through a positive‐feedback loop.

**FIGURE 9:**
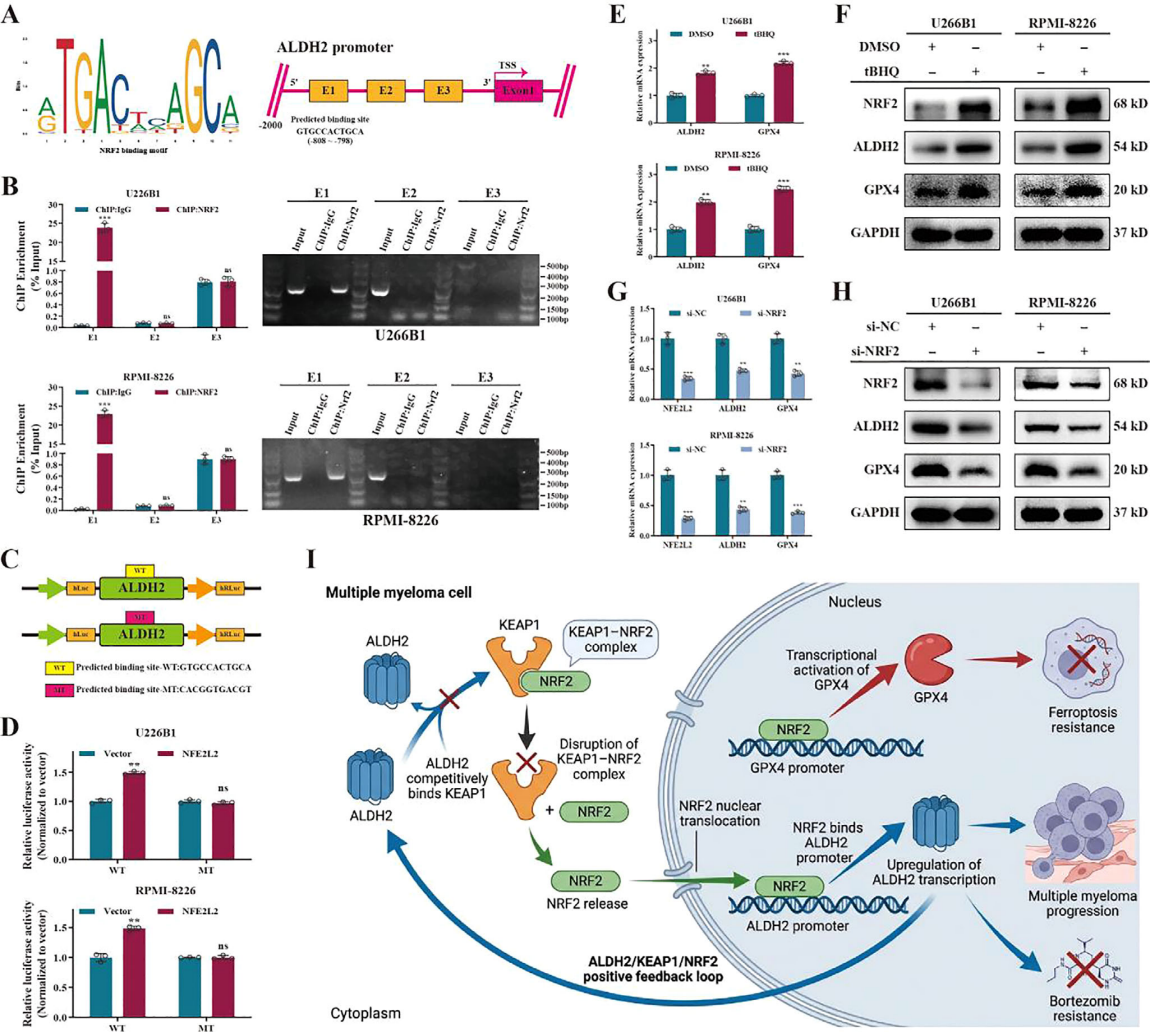
NRF2 promotes ALDH2 transcription in a positive‐feedback model. (A) The NRF2 binding site predicted by the JASPAR database. (B) ChIP analysis of NRF2 binding to ARE in the ALDH2 promoter in MM cells, and then qPCR products were confirmed by nuclear acid electrophoresis. (C) Schematic illustration displayed the dual‐luciferase reporter vectors with wild‐type (Wt) and mutant‐type (Mut) ALDH2 binding sites. (D) Luciferase activity assays assessing the activation of the ALDH2 promoter in MM cells. (E, F) The changes in NRF2, ALDH2, and GPX4 expression levels were analyzed after treated with tBHQ (100 µm). (G, H) After knockdown NRF2, the changes in NRF2, ALDH2, and GPX4 expression levels were analyzed. (I) Proposed model of the relationship between ALDH2 and NRF2 in MM cells. The data are shown as the mean ± SD, ^ns^p>0.05, ^**^ *p* < 0.01, ^***^ *p* < 0.001, and all above experiments have been repeated for three times.

## 3 Discussion

Modulation of ferroptosis has emerged as a promising anticancer strategy. Here, we show that ALDH2, which is overexpressed in MM, competitively binds to KEAP1 and stabilizes NRF2 by preventing its ubiquitin‐proteasome‐mediated degradation to promote the nuclear translocation of NRF2 and subsequent upregulation of GPX4, thereby regulating ferroptosis, proliferation and BTZ resistance. Furthermore, we confirmed that NRF2 binds to the ALDH2 promoter to regulate its transcription. Taken together, our findings suggest that ALDH2 and NRF2 participate in a positive feedback loop that suppresses ferroptosis and maintains BTZ resistance (Figure 9I).

Recent studies have reported that ALDH2 is upregulated in various types of cancer, and its altered expression is closely associated with poor prognosis and chemotherapy resistance [20]. However, the role of ALDH2 in MM and its underlying mechanism has not been reported. Here, we identified that ALDH2 is significantly upregulated in MM, and its high expression is associated with MM progression. Furthermore, we found that the high expression of ALDH2 is associated with poor clinical outcome and BTZ resistance in MM. In addition, the in vitro and in vivo experiments validated that the MM cells with ALDH2‐high expression exhibit markedly increased proliferation activity, BTZ resistance, and ferroptosis resistance. In summary, our findings indicate that ALDH2 acts as an oncogene in MM and serves as a potential therapeutic target for overcoming BTZ resistance and inducing ferroptosis.

Ferroptosis, a non‐apoptotic form of cell death characterized by lipid peroxidation of cellular membranes, has gained considerable attention due to its promising potential as a therapeutic target in overcoming chemoresistance [27, 28, 29]. The tumor microenvironment of MM contains elevated levels of iron, which is actively imported by MM cells for sustaining high rates of DNA replication, nucleotide biosynthesis, and ATP production. However, excessive iron accumulation can cause ferroptosis in MM cells, suggesting that iron dependency represents vulnerability for anti‐MM chemotherapy [25]. Several studies have shown that targeting ferroptosis improves the therapeutic effect of MM [25, 30, 31, 32]. Yan et al. discovered that targeting STK17B kinase activates ferroptosis in BTZ‐resistant MM cells by weakening STAT3 signals, thus significantly increasing the sensitivity of MM cells to BTZ [25]. Li et al. reported that targeting COQ2 by 4‐CBA enhanced the sensitivity of BTZ‐resistant MM cells to RSL3 [30]. Thus, elucidating the underlying mechanism of ferroptosis regulation is essential for facilitating ferroptosis and suppressing BTZ resistance, thereby providing valuable insights into therapeutic resistance in MM.

The KEAP1‐NRF2 pathway is a recognized signaling mechanism that regulates ferroptosis [13]. NRF2 is a crucial regulator of antioxidant gene expression, including GPX4, SLC7A11, and HO‐1, through its interaction with AREs [13, 15]. Abnormal NRF2 activation is linked to poor prognosis and chemoresistance in various cancers [33]. This study shows that ALDH2 functions as a key oncogene in MM by modulating the KEAP1‐NRF2 pathway activation. The regulation of NRF2 signaling is managed by a precisely calibrated control system. Under homeostatic conditions, homodimeric KEAP1, acting as a substrate adaptor for an E3 ubiquitin ligase, facilitates the ubiquitination and degradation of NRF2 [14]. Consequently, NRF2 protein levels can be influenced by either destabilization of the KEAP1‐NRF2 complex or decreased KEAP1 expression. Various regulators have been identified to affect the KEAP1‐NRF2 complex, resulting in persistent NRF2 activation. Nestin binds to the Kelch domain of KEAP1, competing with NRF2 and preventing its degradation, which enhances the production of antioxidant enzymes [34]. iASPP reduces NRF2 ubiquitination by competing with NRF2 for KEAP1 binding through a DLT motif [35]. FAM129B, an antioxidative protein, competes with NRF2 for KEAP1 binding through its DLG and ETGE motifs [36]. DDRGK1 competitively binds to the Kelch domain of KEAP1, obstructing NRF2's interaction with CUL3 and preventing its degradation. The deletion of DDRGK1 leads to NRF2 protein degradation, reducing the expression of downstream antioxidant proteins and consequently increasing ROS production [37]. Consistent with previous research, our findings indicate that ALDH2 competitively binds to KEAP1, thereby obstructing the NRF2‐KEAP1 interaction and preventing NRF2 degradation. ALDH2 knockdown leads to NRF2 protein degradation and reduces GPX4 expression, triggering ferroptosis. Therefore, a connection seems to exist between ALDH2 and ferroptosis.

The expression of ALDH2 is a tightly regulated process. Recent studies have revealed several transcription factor‐dependent and epigenetics‐dependent mechanisms that are responsible for regulating ALDH2 expression in tumorigenesis development. As demonstrated by Wang et al. reported that ALDH2 expression is cooperatively regulated by the histone methyltransferase EHMT2 and the transcription factor NFYA, that this regulation correlates with the paclitaxel resistance of lung cancer [21]. Inhibition of EZH2 enhances ALDH2 expression and promotes NK cell‐induced differentiation and apoptosis in muscle‐invasive bladder cancer [38]. NRF2 is known to function as a transcription factor for certain oncogenes, including Klf948, p62/SQSTM134, Nestin, and ATF349 [34], alongside its role in regulating common antioxidant genes. Interestingly, we found that treatment with the NRF2 activator tBHQ increased the expression of ALDH2 in MM cells. We proposed that NRF2 activation might regulate the oncogene ALDH2. Upon examining the ALDH2 gene promoter for transcription factor‐binding sites, we discovered a potential ARE sequence close to the gene's transcriptional start site. This study demonstrates that ALDH2 expression is increased via the NRF2‐ARE signaling pathway. Meanwhile, knockdown of NRF2 reduced the expression of ALDH2, suggesting that ALDH2 was a downstream target gene of NRF2. These findings enhance our comprehension of how NRF2 activation influences ALDH2. We highlight a positive‐feedback loop between ALDH2 and NRF2, which mediates ferroptosis and sustains BTZ resistance in MM.

## 4 Conclusions

Collectively, our findings reveal that an ALDH2‐NRF2 positive‐feedback loop suppresses ferroptosis and sustains BTZ resistance in MM, positioning this axis as a candidate target for overcoming chemoresistance. Given that ALDH2 is overexpressed in several other cancers, whether targeting the ALDH2/KEAP1/NRF2‐GPX4 axis can enhance chemotherapy response beyond MM is a hypothesis that merits further investigation.

## 5 Experimental Section

### 5.1 Patients and Clinical Samples

The RNA‐expression profiles and clinical information of two MM cohorts, including GSE5900 and GSE68871, were retrieved from the GEO database (http://www.ncbi.nlm.nih.gov/geo/). The RNA‐expression profiles and the half‐maximal dosage effect (IC50) values for MM cell lines were downloaded from the CCLE database (https://portals.broadinstitute.org/ccle). Log2 transformation was conducted to normalize the gene expression profiles. This study was approved by the Institutional Review Board and the Ethics Committee of Sun Yat‐sen University Cancer Center (SYSUCC). The clinical information and BM samples (28 normal BM samples from patients with non‐hematological malignancies and 94 MM patients) were retrospectively collected from SYSUCC (approval ID: B2024‐004‐01). Written informed consent was procured from all study participants. Our research was performed following the guidelines of the Declaration of Helsinki.

### 5.2 Reagents

Bortezomib (BTZ, S1013), MG132 (S2619), RSL3 (S8155), Z‐VAD‐FMK (Z‐V‐F, S7023), necrostatin‐1 (Nec‐1, S8037), Bafilomycin‐A1 (Baf1, S1413), Ferrostatin‐1 (Fer‐1, S7243), ML385 (S8790) and tBHQ (S4990) were obtained from Selleck. Puromycin (HY‐B1743) and Cycloheximide (HY‐12320) were obtained from MedChemExpress. Fast Silver Stain Kit (P0017S), Trypan Blue Staining Cell Viability Test Kit (C0011), and DNA/RNA Loading Buffer (R0211) were purchased from Beyotime. Normal goat serum (ZLI‐9056) and Universal Detection Kit (PV‐6000) were purchased from ZSGB‐BIO. Hematoxylin Staining Solution (G1080) and DAB Substrate Kit (DA1010) were purchased from Solarbio. Universal Blocking and Antibody Dilution Buffer (GF1600) was purchased from Genefist.

### 5.3 Mice and Tumor Models

Female BALB/c nude mice, obtained from Charles River, were raised under standard specific‐pathogen‐free conditions for the mouse xenograft models. Then they were randomly grouped and injected with the designated cell lines. To generate stably overexpressing xenograft models, U266B1 (Approximately 1 × 10^7 ^cells) stably overexpressing ALDH2 or corresponding control cells were injected into the right flanks of 8‐week‐old female BALB/c nude mice. After allowing tumors to grow for one week, mice received either PBS or BTZ (0.5 mg/kg) twice weekly, or RSL3 (5 mg/kg) every other day, or ML385 (30 mg/kg) daily Monday to Friday for a duration of four weeks. Subsequent tumor development was monitored every other day. Finally, tumors were collected for immunohistochemistry. Animal procedures received approval from the Ethical Committee of SYSUCC (approval ID L102012021110B) and adhered to the National Institutes of Health animal care and ethical guidelines. Following the experiment, the mice were euthanized, and the xenografts were collected for weight measurement and further analyses.

### 5.4 Cell Culture Experiments

The U266B1 and RPMI‐8226 cell lines were obtained from the American Type Culture Collection (ATCC). Cells were cultured in RPMI‐1640 medium (C11875500BT, GIBCO) with 10% fetal bovine serum (10270106, GIBCO), 100 IU/mL penicillin, and 100 mg/mL streptomycin (15140122, Thermo Fisher Scientific) in a 37°C humid chamber with 5% CO2.

### 5.5 Plasmid Transfection and Small Interfering RNA (siRNA)

The overexpression plasmids for ALDH2, GPX4, and NRF2 were obtained from Guangzhou Youming Technology Co., Ltd. siRNA‐ALDH2 and siRNA‐NRF2 (GenePharma) were used to knock down ALDH2 and NRF2, respectively. The sequences of the siRNAs were provided in Table S1. Transfection was conducted with Lipofectamine 3000 Transfection Reagent (L3000001, Thermo Fisher Scientific) according to the instructions of the manufacturer.

### 5.6 RNA Extraction and Quantitative Real‐time PCR (qPCR)

Total RNA was extracted using RNA‐Quick Purification Kit (RN001, ES Science), and then a reverse transcription reaction was carried out using Premix PrimeScript RT complex (RR036A, Takara). Real‐time PCR was conducted on a CFX96 system (Bio‐Rad) utilizing TB Green Premix Ex Taq (RR420A, Takara). Each gene was analyzed in triplicate to ensure reproducibility. Table S2 contains a list of premiers.

### 5.7 Malondialdehyde (MDA) and Lipid Peroxidation Assay

The Lipid Peroxidation MDA Assay Kit (S0131, Beyotime) was employed to quantify MDA levels according to the manufacturer's instructions. Lipid peroxidation was evaluated with C11‐BODIPY 581/591 (D3861, Thermo Fisher Scientific). Cells were exposed to C11‐BODIPY in culture medium at 37°C for 30 min. Subsequently, CytExpert software was used for data analysis.

### 5.8 Western Blot Analysis

Cell lysate for immunoblotting was prepared using RIPA buffer (P0013B, Beyotime) with added PMSF (ST507, Beyotime) and protease inhibitors (K1007‐1E, APExBIO). The cell lysate underwent centrifugation at 10 000 × g for 10 min at 4°C to eliminate cell debris. Following the assessment of total protein concentration with the BCA Protein Assay Kit (PH0326, Phygene), 30 µg of protein was denatured, separated via SDS/PAGE, and transferred to a PVDF membrane (ISEQ00010, Millipore). The target protein was immunoblotted with the specific antibody. Refer to Table S3 for the antibodies utilized. Chemiluminescent substrate (G3308, GBCBIO) was used for detecting the signaling intensity. The Nuclear and Cytoplasmic Protein Extraction Kit (P0028, Beyotime) was used to separate cytoplasmic and nuclear proteins.

### 5.9 Co‐Immunoprecipitation (Co‐IP)

To explore the interaction of endogenous proteins, cells were transfected with or without the indicated plasmid for specific experimental purpose. The cells were lysed in IP lysis buffer (P0013, Beyotime) containing protease inhibitors, and the extract was purified by centrifugation at 10 000 × g for 10 min at 4°C. Then the supernatant was incubated with the indicated antibody or a control IgG antibody derived from the same species at 4°C overnight, followed by incubation with Protein A/G Magnetic Beads (HY‐K0202, MedChemExpress) for 2 h at 4°C. The beads were washed three times with IP lysis buffer supplemented with protease inhibitors, and heated to 100°C for 10 min with 1 × SDS. Finally, western blot was conducted following established protocols. The antibodies used for Co‐IP were listed in Table S3.

### 5.10 Ubiquitination Assay

Cells were transfected with the specified plasmid for various experimental purposes, and endogenous NRF2 ubiquitination was analyzed after treatment with MG132 (25 µm) for 4 h to prevent proteasomal degradation. Finally, Co‐IP was performed using NFE2L2 Polyclonal antibody (16396‐1‐AP, Proteintech) and Ubiquitin antibody (3936T, CST) according to standard protocols.

### 5.11 Chromatin Immunoprecipitation (ChIP) Assay

The ChIP assay followed the protocol provided by the Pierce Magnetic ChIP Kit (26157, ThermoFisher). NRF2 (D1Z9C) XPRabbit mAb antibody (12721S, CST) and Rabbit IgG (B900610, Proteintech) were used for the ChIP assay. The DNA underwent purification and qPCR analysis to assess NRF2 binding to the ALDH2 promoter. Refer to Table S4 for the qPCR primer sequences.

### 5.12 EdU and Soft Agar Colony‐Forming Cell Assay

EdU assays were conducted using the EdU Cell Proliferation Kit with Alexa Fluor 594 (C0078S, Beyotime), and cell viability was calculated by the EdU staining positive rate in accordance with the instructions of the kit. In the soft agar colony formation assay, a base layer comprising 0.6% agar (BY‐R0100, Biowest) combined with complete medium was prepared in 6‐well plates and left to solidify at room temperature. Subsequently, cells were suspended in 0.35% agar containing complete medium and seeded onto the base layer at a density of 3000 cells per well. Plates were maintained at 37°C in a humidified 5% CO_2_ environment for 14 days. To prevent desiccation during incubation, 200 µL of complete medium was added every three days. After colony formation, wells were stained with 0.005% crystal violet (548‐62‐9, Solarbio) for 30 min before they were photographed.

### 5.13 Construction of Reporter Plasmid and Luciferase Assay

The wild‐type (Wt) or mutation (Mut) ALDH2‐ARE (ARE‐like site) was cloned into the pGL4.10‐basic luciferase reporter plasmid. The indicated cells (3  × 10^3^) were plated in 24‐well plates and co‐transfected with the designated plasmids and the pRL‐TK Renilla control. Firefly and Renilla signals were quantified 24 h later through the dual‐luciferase reporter gene assay kit (11402ES60, Yeasen) following the manufacturer's guidelines.

### 5.14 Cell Viability

The MM cells were seeded (10^4^ cells) in a 96‐well plate, and then the cells were treated with the specified compound for 48 h, followed by the addition of CCK‐8 reagent (CK04, Dojindo) and continued incubation for 2 h. Optical density at 450 nm was then determined through a microplate reader.

### 5.15 Flow Cytometry‐Based Detection of Apoptosis

Following the specified procedure, MM cells were treated with the indicated compound for 24 h. Subsequently, MM cells were collected in 500 µL Binding Buffer, and then stained with 5 µL FITC‐Annexin V and 5 µL PI (KGA108, KeyGen) for 10 min at room temperature in the dark.

### 5.16 Immunohistochemistry (IHC)

IHC assays were conducted as previously reported [2]. Paraffin sections were incubated with the anti‐Ki‐67, anti‐ALDH2, anti‐GPX4, and anti‐4‐HNE primary antibody against (1:100 dilution). Images were photographed with a Nikon Eclipse 80i microscope. The antibodies used for IHC were listed in Table S3.

### 5.17 Immunofluorescence

Cells were cultured on coverslips, fixed with 4 % paraformaldehyde, and permeabilized with 0.1 % Triton X‐100 (P0096, Beyotime). Subsequently, the cells were incubated with an appropriate primary antibody at 4°C overnight, and then incubated with the secondary fluorescent antibody at room temperature for 1 h in the dark. Finally, the cells were sealed with fluoroshield mounting medium containing 4’, 6‐diamidino‐2‐phenylindole (DAPI) (P0131, Beyotime). Images were acquired using a fluorescence microscope (Olympus, Tokyo, Japan). The antibodies used for immunofluorescence were listed in Table S3.

### 5.18 Establishment of Drug‐Resistant Cell Lines

BTZ‐resistant MM cell lines were developed by exposing parental cells to sublethal doses of bortezomib until they exhibited a fivefold increase in resistance compared to the original cells. BTZ was refreshed every 3 days during cell passaging. Resistance was confirmed by a significant rise in IC50 value. The resistant cells were then collected for further analysis.

### 5.19 Statistical Analysis

All experiments were performed at least three times, and data were presented as means ± standard deviation (SD) unless otherwise specified. Unpaired Student's t test was used to analyze the differences between the two groups. Comparisons among multiple groups were performed using one‐way ANOVA. When all groups were compared with the control group, Dunnett's multiple comparisons test was applied; for comparisons between all pairs of groups, Tukey's multiple comparisons test was used. Survival curves were described by Kaplan‐Meier plots and compared with the log‐rank test. Pearson's correlation coefficient was used to analyze the relationship between NRF2 and ALDH2 expression levels. GraphPad Prism 9 Software was employed for statistical analysis. A two‐sided P‐value below 0.05 was deemed statistically significant. Significance levels are denoted as ^*^ *p* < 0.05, ^**^ *p* < 0.01, and ^***^ *p* < 0.001.

## Author Contributions

RNS, CCL, XPD, JDX, and WJL conducted the experiments and data analysis. BY corrected the language writing. CLS and LRL provided assistance in animal experimentation and data collection. LL, HW and HLT designed and supervised the study. RNS, CCL and XPD wrote the manuscript. All authors have read and approved the final manuscript.

## Conflicts of Interest

The authors declare no conflicts of interest.

## Supporting information




**Supporting File 1**: advs78403‐sup‐0001‐SuppMat.docx.

**Supporting File 2**: advs78403‐sup‐0002‐SuppMat.docx.

## Data Availability

The data that support the findings of this study are available on request from the corresponding author. The data are not publicly available due to privacy or ethical restrictions.
