# Supplementary material for: ALDH2 Suppresses Ferroptosis and Promotes Bortezomib Resistance in Multiple Myeloma Through the KEAP1‐NRF2 Feedback Loop
Source: Adv Sci (Weinh). 2026 Oct 28:e78403. Online ahead of print. doi: 10.1002/advs.78403 (PMC13715670; doi:10.1002/advs.78403)
Supplement: Supplementary file 1 [file ADVS-9999-e78403-s001.docx]

**Fig.S1. (A)** ALDH2 protein expression was elevated in CD138+ plasma cells derived from BTZ‑resistant patients relative to BTZ-sensitive patients. (**B**) qPCR analysis was employed to measure the expression levels of ALDH2 in BTZ-resistant cells and BTZ-susceptible cells. (**C**) ALDH2 expression was progressively up‑regulated during the establishment of BTZ‑resistant MM cell lines. The data are showed as the mean ± SD, ***P < 0.001 and all above experiments have been repeated for three time.

**Fig. S2.** (**A**) Heatmap indicating the upregulated and downregulated genes between si-NC (negative control) and si-ALDH2 MM cells as detected by RNA-seq with three biological duplicates. (**B**) Volcano plot showing differentially expressed genes based on absolute fold change >1 and FDR < 0.05. (**C**) RSL3 induced cell death could not be reversed by apoptosis inhibitor Z-VAD-FMK (Z-V-F, 10 μM), necroptosis inhibitor necrostatin-1 (Nec-1, 1 μM) and autophagy inhibitor Bafilomycin-A1 (Baf1, 50 nM). The data are showed as the mean ± SD, ^ns^p＞0.05, *P < 0.05, **P < 0.01, ***P < 0.001 and all above experiments have been repeated for three time.

**Fig. S3. ALDH2 knockdown attenuates BTZ resistance of MM cells by inducing ferroptosis. (A)** Cell death of ALDH2 suppression in BTZ-treated (10 nM) U266B1R and RPMI 8226R cells upon treatment with three inhibitors, namely, specific inhibitor of ferroptosis ferrostain-1 (Fer-1), apoptosis inhibitor (Z-V-F) and autophagy inhibitor (Baf1). **(B)** Flow cytometry was utilized to detect the lipid peroxidation levels in ALDH2-depleted U266B1R and RPMI 8226R cells under BTZ treatment. **(C)** Flow cytometry was utilized to detect the Cleaved-caspase-3 levels in ALDH2-depleted U266B1R and RPMI 8226R cells under BTZ treatment. (**D-E**) The lipid peroxidation and MDA levels in MM cells under BTZ (10 nM) or/and RSL3 (0.5 μM) treatment. (**F**) MM cells were treated with varying doses of BTZ and RSL3, both individually and in combination, for 48 hours. Cell viability was subsequently assessed using CCK8, with a Combination Index (CI) value of BTZ and RSL3 less than 1 indicating synergy. The CI was determined by CompuSyn software program. The data are showed as the mean ± SD, ^ns^p＞0.05, *P < 0.05, **P < 0.01, ***P < 0.001 and all above experiments have been repeated for three time.

**Fig. 4.** (**A**) RSL3 induced cell death could be overwhelmingly reversed by GPX4 overexpression. (**B**) Lipid peroxidation was measured by BODIPY™ 581/591 C11 staining in MM cells subjected to RSL3 (0.5 μM). The data are showed as the mean ± SD, ^ns^p＞0.05, ***P < 0.001 and all above experiments have been repeated for three time.

**Fig. S5.** (**A**) Mass spectrum pictures suggesting that KEAP1 was enriched in ALDH2 binding proteins. (**B**) Co-IP assay conducted using anti-ALDH2. Samples were separated by silver staining followed by mass spectroscopy in RPMI 8226 cells. (**C**) The sequence of STGE short-linear motif in human ALDH2 sequence. (**D**) Human ALDH2 crystal structure. (**E**) The predicted interaction between ALDH2 and KEAP1. (**F**) Statistical quantification of the intensity of NRF2 protein that binds to KEAP1. (**G**) ALDH2 reduced the interaction between NRF2 and KEAP1. ALDH2-overexpression and/or KEAP1-overexpression MM cells were treated with 25 μM MG132 for 4 h. Cell lysates were immunoprecipitated with an anti-KEAP1 antibody and blotted with anti-NRF2 antibody. The data are showed as the mean ± SD, **P < 0.01 and all above experiments have been repeated for three time.

**Fig. S6. (A)** Western blotting analysis was employed to measure the expression levels of NRF2, ALDH2 and GPX4 in BTZ-resistant cells and BTZ-susceptible cells. **(B)** MM cells transfected with ALDH2-overexpression and control plasmids were left untreated or treated with ML385 and incubated with Doxycycline (Dox, 1ug/mL) for the indicated time periods. Lysates were analyzed by Western blotting. (**B**) Statistical quantitation of the NRF2 protein intensity. **(C)** Dose-response curves of BTZ in MM cells measured by CCK-8 assay. (**D**) MM cells were treated with varying doses of BTZ and ML385, both individually and in combination, for 48 hours. Cell viability was subsequently assessed using CCK8, with a CI value of BTZ and ML385 less than 1 indicating synergy. (**E**) Left panel, representative image of tumors from xenografted mice treated with BTZ and/or ML385 after 5 weeks (n = 5 each group); right panel, the weight of tumors was measured after surgical dissection. (**F**) The volumes of tumor were measured every 5 days and the growth curves of each group were drawn. The data are showed as the mean ± SD, *P < 0.05, ***P < 0.001 and all above experiments have been repeated for three time.

**Fig. S7.** Relative cell viability was assessed *via* CCK-8 in CD138+ MM cells treated with BTZ (50 nM), and/or RSL3 (1 μM), and/or ML385 (100 μM) for 24 h. The data are showed as the mean ± SD, ***P < 0.001 and all above experiments have been repeated for three time.
