# Supplementary material for: ALDH2 Suppresses Ferroptosis and Promotes Bortezomib Resistance in Multiple Myeloma Through the KEAP1‐NRF2 Feedback Loop
Source: Adv Sci (Weinh). 2026 Oct 28:e78403. Online ahead of print. doi: 10.1002/advs.78403 (PMC13715670; doi:10.1002/advs.78403)
Supplement: Supplementary file 2 [file ADVS-9999-e78403-s002.docx]

**Supplementary Table S1**

| Gene |  | Sequence (5’-3’) |
| --- | --- | --- |
| si-ALDH2-1 | sense | GGCAACUGGAAACGUGGUUTT |
|  | antisense | AACCACGUUUCCAGUUGCCTT |
| si-ALDH2-2 | sense | CCUCAAAUGUCUCCGGUAUTT |
|  | antisense | AUACCGGAGACAUUUGAGGTT |
| si-NRF2 | sense | GCCCAUUGAUGUUUCUGAUTT |
|  | antisense | AUCAGAAACAUCAAUGGGCTT |
| si-NC | sense | UUCUCCGAACGUGUCACGUTT |
|  | antisense | ACGUGACACGUUCGGAGAATT |

**Supplementary Table S1. The sequence of siRNA used in this study.**

**Supplementary Table S2**

| Primer name | Sequence (5’-3’) | Tm |
| --- | --- | --- |
| GAPDH Forward | CTGACTTCAACAGCGACACC | 59.14 |
| GAPDH Reverse | TGCTGTAGCCAAATTCGTTGT | 58.77 |
| ALDH2 Forward | ATGGCAAGCCCTATGTCATCT | 59.22 |
| ALDH2 Reverse | CCGTGGTACTTATCAGCCCA | 59.17 |
| GPX4 Forward | CAGTGAGGCAAGACCGAAGT | 59.97 |
| GPX4 Reverse | CTGCTTCCCGAAGTGGTTAC | 58.56 |
| NQO1 Forward | GAAGAGCACTGATCGTACTGGC | 61.05 |
| NQO1 Reverse | GGATACTGAAAGTTCGCAGGG | 58.72 |
| KEAP1 Forward | GTGTCCATTGAGGGTATCCACC | 60.42 |
| KEAP1 Reverse | GCTCAGCGAAGTTGGCGAT | 61.1 |
| NFE2L2 Forward | TCCAGTCAGAAACCAGTGGAT | 58.66 |
| NFE2L2 Reverse | GAATGTCTGCGCCAAAAGCTG | 60.99 |

**Supplementary Table S2. The sequence of primers for qPCR assay used in this study.**

**Supplementary Table S3**

| Antibody | Brand | Cat. | Application |
| --- | --- | --- | --- |
| GPX4 Monoclonal antibody | Proteintech | 67763-1-IG | WB, IHC |
| NFE2L2 Polyclonal antibody | Proteintech | 16396-1-AP | WB, IP |
| KEAP1 Monoclonal antibody | Proteintech | 60027-1-IG | WB, IP |
| GAPDH Polyclonal antibody | Proteintech | 10494-1-AP | WB |
| ALDH2 Polyclonal antibody | Proteintech | 15310-1-AP | WB, IHC, IP |
| Histone-H3 Rabbit Polyclonal Antibody | Proteintech | 17168-1-AP | WB |
| Ubiquitin Polyclonal antibody | Proteintech | 10201-2-AP | WB, IP |
| 4-Hydroxynonenal Monoclonal antibody | Proteintech | 68538-1 | IHC |
| Ki-67 Polyclonal antibody | Proteintech | 27309-1-AP | IHC |
| Rabbit IgG | Proteintech | B900610 | IP, ChIP |
| CoraLite488-conjugated Goat Anti-Rabbit IgG(H+L) | Proteintech | SA00013-2 | IF |
| CoraLite594-conjugated Goat Anti-Mouse IgG(H+L) | Proteintech | SA00013-3 | IF |
| CoraLite594-conjugated Goat Anti-Rabbit IgG(H+L) | Proteintech | SA00013-4 | IF |
| HRP-conjugated Affinipure Goat Anti-Rabbit IgG(H+L) | Proteintech | SA00001-2 | WB |
| HRP-conjugated Affinipure Goat Anti-Mouse IgG(H+L) | Proteintech | SA00001-1 | WB |
| NRF2 (D1Z9C) XP®Rabbit mAb antibody | [Cell Signaling Technology](https://www.bing.com/ck/a?!&&p=9a3a48311158e374f55bd1f79c319607c4a56772d98ca98e09757c3a8f73e07aJmltdHM9MTc1NzgwODAwMA&ptn=3&ver=2&hsh=4&fclid=38dcc5d6-cb10-6514-28ce-d1e6ca2864b7&psq=CST%e8%8b%b1%e6%96%87&u=a1aHR0cHM6Ly93d3cuY2VsbHNpZ25hbC5jbi8&ntb=1) | 12721S | IF, ChIP |
| Ubiquitin (P4D1) Mouse mAb | [Cell Signaling Technology](https://www.bing.com/ck/a?!&&p=9a3a48311158e374f55bd1f79c319607c4a56772d98ca98e09757c3a8f73e07aJmltdHM9MTc1NzgwODAwMA&ptn=3&ver=2&hsh=4&fclid=38dcc5d6-cb10-6514-28ce-d1e6ca2864b7&psq=CST%e8%8b%b1%e6%96%87&u=a1aHR0cHM6Ly93d3cuY2VsbHNpZ25hbC5jbi8&ntb=1) | 3936T | WB |

**Supplementary Table S3. Antibodies used in this study.**

**Supplementary Table S4**

| Primer name | Sequence (5’-3’) |
| --- | --- |
| F1 Forward | AGGCAGGAGACTGGTGTGAA |
| F1 Reverse | TCTTGGAAGCAGCGGTTCAG |
| F2 Forward | CTCTCCTTGTTAACTGGGCTC |
| F2 Reverse | GAGGTGAAGGAGATGAACGAG |
| F3 Forward | CCAAGGCGAGCTGCGTT |
| F3 Reverse | AGGGCCACTGAGCCGAC |

**Supplementary Table S4. The sequence of primers for ChIP-qPCR assay used in this study.**
